# Perception in Black and White: Effects of Intonational Variables and Filtering Conditions on Sociolinguistic Judgments With Implications for ASR

**DOI:** 10.3389/frai.2021.642783

**Published:** 2021-07-15

**Authors:** Nicole R. Holliday

**Affiliations:** University of Pennsylvania, Philadelphia, PA, United States

**Keywords:** intonation, sociophonetics, African American English, ethnic identification, automatic speech recognition, speech synthesis

## Abstract

This study tests the effects of intonational contours and filtering conditions on listener judgments of ethnicity to arrive at a more comprehensive understanding on how prosody influences these judgments, with implications for austomatic speech recognition systems as well as speech synthesis. In a perceptual experiment, 40 American English listeners heard phrase-long clips which were controlled for pitch accent type and focus marking. Each clip contained either two H* (high) or two L+H* (low high) pitch accents and a L-L% (falling) boundary tone, and had also previously been labelled for broad or narrow focus. Listeners rated clips in two tasks, one with unmodified stimuli and one with stimuli lowpass filtered at 400 Hz, and were asked to judge whether the speaker was “Black” or “White”. In the filtered condition, tokens with the L+H* pitch accent were more likely to be rated as “Black”, with an interaction such that broad focus enhanced this pattern, supporting earlier findings that listeners may perceive African American Language as having more variation in possible pitch accent meanings. In the unfiltered condition, tokens with the L+H* pitch accent were less likely to be rated as Black, with no effect of focus, likely due to the fact that listeners relied more heavily on available segmental information in this condition. These results enhance our understanding of cues listeners rely on in making social judgments about speakers, especially in ethnic identification and linguistic profiling, by highlighting perceptual differences due to listening environment as well as predicted meaning of specific intonational contours. They also contribute to our understanding of the role of how human listeners interpret meaning within a holistic context, which has implications for the construction of computational systems designed to replicate the properties of natural language. In particular, they have important applicability to speech synthesis and speech recognition programs, which are often limited in their capacities due to the fact that they do not make such holistic sociolinguistic considerations of the meanings of input or output speech.

## Introduction

The questions of whether and how listeners can distinguish Black American and White American voices have been a popular topic in phonetic and sociolinguistic studies over the past 50 years, with implications for both the linguistic understanding of perception as well as issues of social inequality (see for review Thomas and Reaser 2004; Thomas et al., 2010). In general, these studies have found that listeners are fairly adept at distinguishing Black American and White American voices, though the literature has not yet completely established which acoustic parameters may influence listeners’ judgments. In particular, though scholars have posited distinctive patterns of intonation, prosody, and voice quality associated with varieties of African American Language^1^, the specific acoustic characteristics of these varieties are still not well-described. This is a serious lacuna, because as a result of their perceptual salience, intonational features are especially important in the analysis of linguistic profiling, or what noted linguist John Baugh has recently called “Speaking While Black”, the phenomenon by which African Americans experience discrimination, sight-unseen, because their speech may act as an indicator of their race (2015).

As scholars such as Baugh (2000, 2003, 2015) and Thomas and Reaser (2004) have noted, understanding the ways in which listeners make ethnicity judgments is crucial for working against discrimination and linguistic profiling. In his 2015 chapter in the Oxford Handbook of African American Language (OHAAL), Baugh provides ample evidence of this type of discrimination in the courtroom, in housing, and in the workplace. Indeed, though we know THAT listeners make these judgments, the question of precisely HOW still escapes sociolinguists. This understanding is of vital importance due to the fact that until we know how linguistic profiling occurs, we will not be able to provide professionals across industries as well as the public with strategies to recognize and combat this type of discrimination. As Baugh observes, “it is important that those who speak non-dominant dialects or non-dominant languages are aware of their linguistic circumstances, but also the constraints they may face from those who are fluent speakers of surrounding dominant languages and dialects” (768). As a result, linguists have a powerful motivation to better understand the scientific mechanisms that underlie their social judgments about language.

Traditionally, much of the literature that examines the way in which varieties of African American Language are stigmatized has focused on phonological and morphosyntactic differences between AAL varieties and Mainstream U.S. English (MUSE) (Spears 1988; Thomas 2015). However, some research has indicated that speakers can be reliably identified as Black by listeners, even in the absence of non-standard grammatical features (Purnell et al., 1999; Thomas and Reaser 2004; Holliday and Jaggers 2015). In these contexts, even Black speakers who do not use stereotyped variables associated with AAL may still be subject to linguistic profiling and discrimination due to their use of intonational and prosodic features that index Blackness in the minds of listeners. This substantial gap related to the study of intonational features in the literature represents a serious challenge for both linguists and lay people alike, especially given that suprasegmental features are among the elements of speech that are most salient for listeners, even if they are not consciously aware of this fact (Thomas 2015).

Beyond issues related to linguistic profiling by humans in real-life situations, the lack of research on ethnolinguistic variation at the level of intonation also represents a challenge for scholars interested in how to employ listener judgment and production data for computational applications. A number of recent studies have begun to show the limitations of assuming ethnolinguistic homogeneity in language recognition and synthesis programs, and have advocated for addressing the role of several types of bias in NLP applications (Blodgett et al., 2020; Shah et al., 2020). Though the majority of this research has focused on large-scale corpus data such as tweets, the issues may be even more pressing for the analysis of spoken data. Tatman and Kasten (2017) tested effects of talker gender and race on automatic speech recognition in two (ASR) systems: Bing Speech and Youtube automatic captions, and found a significantly higher word error rate for African American talkers than for White ones. In this way, Black American speakers experience linguistic discrimination not just by humans, but also by systems designed to process human language, as systems that do not consider ethnolinguistic variation are more likely to fail them. However, this outcome is not inevitable: Lehr et al. (2014) found that specifically training a discriminative pronunciation model on AAVE data improved the model’s accuracy by 2.1%, showing that with proper data and training, systems can begin to accommodate complex ethnolinguistic variation.

In particular, as speech recognition and speech synthesis systems become more integrated, having a better understanding of the criteria that listeners use when making social judgements under different types of listening conditions thus is important for improving the quality of models. Incorporating information about ethnolinguistic variation may be especially vital for researchers working in the area of speech synthesis. Individuals with medical conditions that impact their speech frequently rely on systems like Speech Generating Devices (SGD), and patients show strong preferences for systems that generate voices that align with their social identities (Crabtree et al., 1990; Creer et al., 2013). To date, few existing systems take into account factors such as ethnolinguistic variation, but having a naturalistic voice output system has been shown to improve the quality of life for patients with these types of conditions (Creer et al., 2013).

The current study begins to address these issues by examining two suprasegmental parameters that have been observed to be involved in such ethnolinguistic variation; pitch accent type and focus marking, as potential loci of information that listeners may use to make ethnicity judgments. By focusing on these two variables, which have been observed to differ between Black and White speakers in production studies (Author 2016; McLarty 2018), linguists may be able to start to pinpoint the intonational variables that influence listener ethnicity judgments. This study also builds on earlier works on production to investigate the relationship between variables that speakers use to perform certain types of racial identity as well as listener judgments of those same variables. It aims to corroborate the observations in earlier studies that have found listeners accurate at judging ethnicity, but also to carefully control the intonational phenomena in the stimuli to investigate the role of those variables in these types of judgments. It also challenges some of the assumptions made in earlier ethnicity judgment studies by showing that intonational contours may be judged differently by listeners when they are exposed to filtered vs. unmodified speech.

The majority of previous studies on ethnic identification exposed listeners to unaltered sound clips that were made in a laboratory setting, and also asked listeners to make judgments in a laboratory setting under ideal listening conditions (cf Thomas and Reaser 2004). One limitation of this methodology is that laboratory listening conditions may differ from the everyday type of listening environments where linguistic profiling happens. The current study’s findings provide further evidence that linguists should consider the potential effects of listening environment since the results indicate that listeners may pattern in opposite ways with respect to ethnicity judgments, depending upon the intonational contours of the stimuli as well as how the stimuli may be filtered. This difference in judgments based on intonation and filtering may be even more important to understand for use in computational systems, given that the corpora employed frequently employ data that has been recorded under imperfect acoustic conditions that may have filtering effects, such as YouTube Videos (Tatman and Kasten 2017). Furthermore, for speech synthesis applications, users necessarily interact with listeners in imperfect acoustic settings, indicating that the creation of more naturalistic synthesized voices must consider the way listeners evaluate speakers in a variety of conditions.

## Perception, Recognition, and Production of Ethnolinguistic Variation

### Perception Studies

As observed by Thomas (2015) and Author (2016), the overall lack of information about the role of suprasegmental features, such as intonation and prosody, in the speech of Black Americans presents an important challenge for researchers. Despite the evidence that prosodic information is highly salient for listeners when making judgments about speaker ethnicity, we still have very little information about how different acoustic parameters may affect these assessments (Purnell et al., 1999). This also presents particular difficulty for inclusive ASR systems; if we do not understand the parameters that listeners use to distinguish voices, we cannot properly evaluate systems that should be able to respond to the variation inherent in large communities of users. In particular, recent research on bias in NLP models reveals tendencies to exclude or stereotype language employed by Black users, leading to communities being not only underserved but also harmed by such systems (Blodgett et al., 2020). Sociolinguistic research on such bias as well as how listeners interpret social properties of voices may be able to help researchers in ASR and synthesis begin to address these inequalities.

In their 2004 study and review of the literature on ethnic identification, Thomas and Reaser discuss 30 studies from across the U.S. that have generally supported the finding that American English speakers are adept at identifying the race of a speaker, even based on hearing a very short sound clip, indicating the role of features beyond the segmental level. Although AAL suprasegmental features (and intonational features in non-standard English varieties overall) have received less attention than other types of phonological or morphosyntactic features, a few scholars have addressed the role of these features in ethnicity judgments. In this vein, there are a number of studies that have approached this type of variation from a production perspective, and others that have addressed it from a perception perspective. In general, the production studies have been more likely to employ methodologies designed specifically to test the variables involved in ethnolect variation, although this can also be observed in some of the perception studies that have conducted posthoc analyses of the variables involved. The section that follows will begin by describing the findings of earlier perception studies, and will then discuss the findings of relevant production studies on listener judgments of ethnicity.

In one of the earliest ethnic identification studies in the U.S., Bryden (1968) analyzed Black and White speakers in Charlottesville, Virginia, and found that ethnicity was correctly identified for 74% of speakers in unfiltered stimuli, and correctly identified for bandpass filtered stimuli approximately 68% of the time. In this study, listeners heard unfiltered recordings of racially matched speakers and then heard the same tapes again that had undergone bandwidth compression using spectral filtration. The stimuli were taken from 35 White and 35 Black children reading the United States’ Pledge of Allegiance. In the filtered listening condition, stimuli were band pass filtered below 1,250 and above 1,750 Hz. Bryden motivates this level of band pass filtering by claiming that a filter between 1,250 and 1,750 Hz is the maximum filtering condition that can be employed without loss of intelligibility. The listeners heard 20 filtered and 20 unfiltered clips each and the listening population included 40 listeners, 20 of whom were Black and 20 of whom were White, and 8 of whom had some previous training in the field of communication sciences and disorders. Bryden’s primary finding was that listeners’ ability to make accurate ethnicity judgments is somewhat degraded in filtered conditions, but that listeners still performed better than chance even in these bandpass filtered conditions, showing the durability of listener judgments even with degraded stimuli.

Building on this work, Koutstaal and Jackson (1971) examined ratings of the voices of 10 male speakers in Ohio, and found that speakers were over 80% accurate in their identifications, though they were somewhat more accurate with White speakers than Black speakers. 26 listeners heard “five negro colloquialisms”^2^ that had been read by 5 Black and 5 White Ohio speakers, and were instructed to simply indicate whether the speaker was Black or White. Results indicated that the White speakers were almost categorically identified as White (4 were identified at 100% accuracy and one at 92% accuracy). However, the authors observed substantially more variation for the Black speakers, with two of the Black speakers identified at 100% accuracy and the others identified at 85, 81, and 69%, respectively. Koutstaal and Jackson (1971) also conducted a posthoc analysis of the samples using spectrograms, specifically examining syllable times, overall speaking time and F0 for each clip. They identified the presence of different contours for Black and White speakers, but concluded that contour shape was not predictive of speaker identification^3^. They also found no consistent differences for syllable duration or any of the other suprasegmental variables that they examined. Ultimately, the authors speculate that listeners may use segmental information rather than intonational information in their judgments, but they do not systematically evaluate these differences.

Lass et al. (1980) conducted a study in which listeners heard sentences from 10 male and 10 female speakers that had been read in an unfiltered condition, low pass filtered at 255 Hz, and high pass filtered at 255 Hz. These filtering conditions were used to attempt to focus on which variables listeners may attune to in their judgments. A low pass filter at 255 has the effect of eliminating vowel formant while retaining fundamental frequency, while a high pass filter at 255 Hz has the effect of focusing listener attention on vowel formants, though the signal still retains traces of F0 information (Thomas 2011:76). Lass et al. (1980) found that listeners correctly identified speaker ethnicity 72% of the time in the unfiltered condition and that identification rates were lower but still reliable for the other conditions, with accuracy rates of 69% for the high pass filtered condition and 60% in the low pass filtered condition. The authors concluded, based on these results, that formants are generally more important than F0 measures for ethnic identification, given the higher accuracy rate with the high pass filter.

In addition to their review of the earlier literature, Thomas and Reaser (2004) also conducted an experiment where they examined ethnic identification with Black and White American speakers from Hyde County, North Carolina, as well as inland regions of North Carolina. In their experiment, 117 listeners rated three different types of clips. In one condition, the clips were unmodified. In the second condition, they were monotonized using KayAnalysis Synthesis Laboratory with F_0_ set at 120 Hz for male speakers and 200 Hz for female speakers, in order to eliminate F_0_-dependent variation. In the third condition, the stimuli were low pass filtered at 330 Hz, in order to preserve intonational information while removing nearly all vowel quality cues. Their results indicated a high level of accuracy for the monotonal treatment among all listeners, and a rate of accuracy close to chance for the low pass filtered conditions. Thomas and Reaser (2004) do note that the filtered stimuli containing prominent subject pronouns were more readily identified than those without such pronouns, indicating that listeners may be relying on at least some intonational information in making their judgments, though they were not specific about what types of intonational contours occurred in these contexts.

Foreman (2000) found that listeners were over 80% accurate in ethnic identification and that those listeners with greater exposure to both White and Black voices were the most accurate. In this study, she tested 20 Black listeners and 19 White listeners on recordings of a script made by 6 Black and 4 White speakers. The stimuli consisted of 54 sentences with “distinctive intonational patterns”, though Foreman is not explicit about what these intonational patterns were. The stimuli were low pass filtered at 900 Hz to partially obscure segmental and voice quality cues in order to specifically test the role of intonation. It is important to note that in contrast with the study conducted by Lass et al., this filter setting at 900 Hz still allows for some formant information as well as a higher level of intelligibility of the signal. Foreman notes that the sentences with “ethnically diagnostic intonation patterns” were most easily identified, though she does not state exactly which contours she tested. Despite the fact that Foreman is not specific about which “dialect specific” intonational contours she employed in the stimuli, she does claim that stimuli with “distinctively” Black intonation are more likely to be correctly identified has having been uttered by a Black speaker, providing evidence for the importance of intonational variation in these judgments. Supporting the patterns also observed by Buck (1968) and Koutstaal and Jackson (1971), Foreman also found that listeners were less accurate in identifying Black speakers than White speakers, a finding that she attributes to the fact that Black speakers may not always employ stereotypical AAL features in every utterance.

Foreman (2000) findings are especially important in light of how the results of these ethnic identification tasks may be important for computational linguistic applications. Foreman posits an expectation that listeners are waiting to hear stereotypical AAL features, and that when they do not, they have lower levels of accuracy in the ethnic identification task. Given that Lehr et al. (2014) were able to improve the accuracy of an ASR model by training it on phonological and morphosyntactic features of AAL, it may be reasonable to hypothesize that such training on intonational features may provide even greater improvements to such models. Since ASR systems can be trained to examine features at all linguistic levels, not just those with stereotypical salience, understanding the role of prosodic variation may allow such systems to improve on listener-ratings, if the systems can be trained to avoid the pitfalls of stereotypes that listeners may experience. In this way, examining the performance of ASR systems at different levels of filtering may also help us better isolate which variables may be more or less salient for human listeners in similar tasks.

### Perception and ASR Systems

With respect to how such ethnicity judgments may affect the performance of speech recognition and synthesis systems, little work has specifically explored how such systems may evaluate ethnic differences between inputs. In fact, not only is there a dearth of literature examining how ASR systems may incorporate sociolinguistic information at any level, there is also very little that directly compares how humans and systems incorporate different acoustic information in ways that may be similar or different from each other. This is a serious problem for researchers across fields, because as Blodgett et al. (2020) observe “work must be grounded in the relevant literature outside of NLP that examines the relationships between language and social hierarchies; without this grounding, researchers and practitioners risk measuring or mitigating only what is convenient to measure or mitigate, rather than what is most normatively concerning” (6). Despite this limitation related to disconnects between linguists and computational researchers and lack of research comparing human and machine performance, some work has begun to address issues related to both managing variable inputs and accounting for noise in ASR systems. Unfortunately, many modern systems rely on proprietary deep learning algorithms for speech recognition and generation, so the properties of these systems are not necessarily transparent. Earlier foundational research, however, has discussed the mechanisms that underlie some of these processes, which will allow us to discuss how systems have addressed variable acoustic inputs.

Linguists and other researchers have long observed the necessity for naturalistic prosody in computational linguistic applications. In their 2010 paper, Vicsi and Szaszák, 2010 address acoustic processing and modelling of the suprasegmental speech properties and find that the addition of prosodic information, in this case F0 and energy, significantly improves word recognition and boundary detection in models for both Hungarian and Finnish. Their system begins with Hidden Markov Model units that they then train and connect to a broader language model. They subsequently use the HMM framework to model prosody and conduct syntactic and/or semantic level processing of the input speech and then used HMMs to model each clause’s prosodic contour. They claim that the addition of prosodic contour modeling increased accuracy; for Hungarian data, word recognition improved by 3.82% with the addition of prosodic information.

In an early work on prosody modeling for ASR, Shriberg and Stolcke, (2004) examine a number of different strategies for incorporating prosodic information into their models. One of their main arguments relates to the fact that computational systems need not necessarily process and manage linguistic input the way that human coders do. In fact, they argue that computational systems should be modeled “directly in a statistical classifier—without the use of intermediate abstract phonological categories, such as pitch accent or boundary tone labels. This bypasses the need to hand-annotate such labels for training purposes, avoids problems of annotation reliability, and allows the model to choose the level of granularity of the representation that is best suited for the task” (2). Unfortunately, this creates a significant difference from how linguists interested in human speech model prosodic information, making the two approaches difficult to compare directly. However, these authors do provide important information about the criteria that many models are based on, noting that their method is based on contour classification on both syntactic and semantic models. In particular, they note that the most successful models that they observe “extract features from a forced alignment of the transcripts (usually with phone-level alignment information), which can be based on either true words, or on (errorful) speech recognition output…This yields a rich inventory of “raw” features reflecting F0, pause and segment durations, and energy (2). Though these models rely on statistical classifiers as opposed to the phonological categories used by non-computational researchers, the features that their model incorporates overlap significantly with the features that human coders use to do prosodic labelling. To date, I have found no research that directly compares human coders and statistical models for prosody, but Shreiber and Stolcke’s findings provide support for the claim that ASR models may be trained to use the same type of phonetic criteria for prosodic labelling that human coders use (Cole and Shattuck-Hufnagel 2016). As a result, a better understanding of the phonetic cues that may differ between ethnolinguistic communities has the potential to enhance the accuracy of ASR systems as well.

As the current study is interested in how humans make ethnicity judgments under different listening conditions and how this may compare with computational systems, how ASR systems perform under noisy conditions is another important point of consideration. Li et al. (2014) provide a comprehensive overview of the literature on noise-robust ASR as well as a useful comparison that clearly articulates advantages and disadvantages of various popular models. While a full discussion of the five types of models they compare is beyond the scope of this review, they do provide some important points that are especially relevant to the current study. In particular, they compare systems that employ five different types of attributes: “feature vs. model domain processing, explicit vs. implicit distortion modeling, use of prior knowledge about distortion or otherwise, deterministic vs. uncertain processing, and joint vs. disjoint training” (768). Though many modern systems rely on neural-network based methods or CD-DNN-HMM, a number of the older methods continue to provide the basis for their assumptions; this is particularly the case for explicit distortion modeling. Li et al. argue that “noise, channel, and speaker factors may already be well normalized by the complex nonlinear transform inside the DNN. However, this does not mean that the noise-robustness technologies are not necessary when used together with CD-DNN-HMM” (771). This indicates that the authors believe that ASR can be improved when models are given explicit training on the speech context, including information about the speaker, which may also include the type of sociolinguistic information available to human listeners. Having examined both how humans use acoustic information to make judgments and how ASR systems use such information in their models, we now turn to the question of how the same acoustic variables have been examined in studies that focus on how humans produce speech.

### Production Studies and the Tone and Break Index System

Though production studies on ethnicity and suprasegmental variables have also been somewhat rare, several have focused on observing systematic differences between Black and White speakers in a variety of speech settings. Unfortunately, few have tested whether these differences in production are truly salient for listeners, which is an especially important consideration for speech synthesis.

In terms of the specific intonational features that may differ between MUSE and AAL patterns, there have been only a few studies that have examined this question in a modern framework. Starting in the 1980’s, intonational phonologists and phoneticians began to employ the modern Tone and Break Index transcription system for General American English (Pierrehumbert 1980; Beckman and Pierrehumbert 1986, cf ; Beckman et al., 2005). This system consists of an inventory of pitch contours (tones) and phrase boundaries (breaks) and is in widespread use in the modern literature on intonational phonology (ibid, Thomas et al., 2010; Thomas 2015, inter alia). It is especially useful for systematically examining variation and providing a consistent framework for labeling intonational contours and phrase boundaries, and so the majority of intonation studies published in the U.S. in the last 30 years have employed the ToBI annotation conventions. The full guidelines for TobI labelling can be found in Beckman and Ayers (1997), though for the current study and those discussed below, the primary points of interest in the ToBI system are pitch accents and boundary tones, which consist of a number of combinations of high (H) and low(L) tones, which can generally be seen in the shape of the F0 contour on a spectrogram. In English, pitch accents can only occur on stressed syllables, and they are the main cue to prominence. They are typically realized with a combination of some type of F0 movement as well as other cues such as longer duration and higher intensity. The pitch accents of interest for the current study will be discussed in greater detail in the methodology section, though understanding the basics of the framework is necessary for interpreting the findings of Jun and Forman, (1996), McLarty (2018), and Author (2016) which are discussed below.

Jun and Forman (1996) provide the first formal analysis of AAL intonation based on this autosegmental metrical model and the ToBI system (Pierrehumbert 1980). Jun and Forman (1996) recorded 7 same-race dyads (5 Black and 2 White), enacting the same scripted dialogue. They found that in general, the Black speakers (who were all speakers of AAE), employed wider pitch range and higher pitch at phrase boundaries than the White MUSE speakers. Specifically, they were interested in the patterning of Yes-No questions, and found that AAE speakers appeared to have a different pattern than the MUSE speakers, such that the AAE speakers were more likely to use a low tone followed by a high flat tone (L* H-L% in ToBI) while MUSE speakers use a low tone followed by a rising high boundary (L* H-H% in ToBI) though the differences between the two speaker groups were less consistent for declaratives and Wh-questions. This study represented an important first step for systematically analyzing the differences between MUSE and AAL using modern intonational techniques, though its design and focus on phrase boundaries and Yes-No questions limits its applicability for testing listener judgments of ethnicity using declaratives and naturalistic speech.

McLarty (2018) also attempted to quantify the specific differences between Black and White speakers with respect to intonational variables, using the ToBI framework. McLarty studied phenomena related to differences in pitch accent types, using the ex-slave recordings previously employed in linguistic research by Bailey et al., (1991) as well as contemporary speakers from Raleigh, North Carolina, McLarty found that African Americans in both the ex-slave and modern recordings used a greater incidence of the L+H* (low target followed by high target with prominence, in the same syllable) pitch accent as compared to the H* (high) pitch accent, when compared to the MUSE speakers in his study. McLarty argues that this may provide further evidence for a generally different pattern of use of intonational contours between MUSE and AAL speakers, though he also did not test the salience of these observed differences for listeners.

**FIGURE 1:**
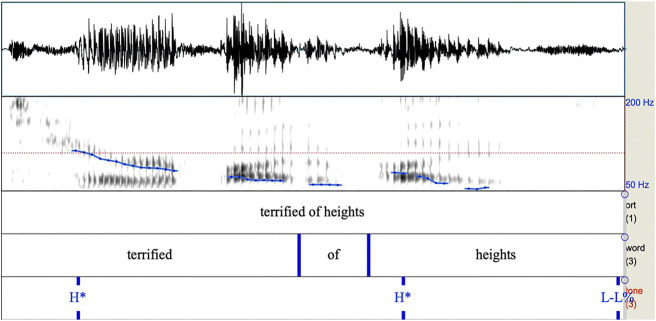
Spectrogram of intonational phrase from Author (2016) containing two H* pitch accents followed by a L-L% boundary tone. Note the high F0 on the first syllable of terrified, and the rise on the first syllable of “heights”.

**FIGURE 2:**
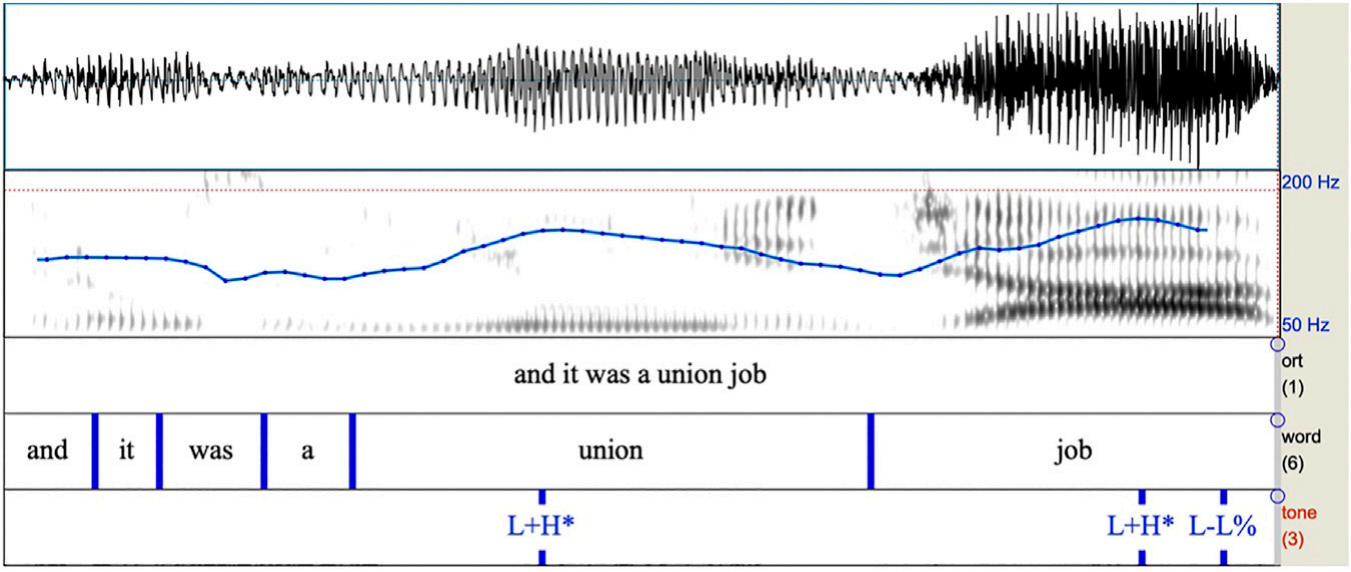
Spectrogram of intonational phrase from Author (2016) containing two L+H* pitch accents followed by a L-L% boundary. Note the F0 fall and subsequent rise before the first syllable of “union”. The same pattern occurs for the word “job”.

In my earlier study, Author (2016), I examined casual speech data from young men with one Black parent and one White parent, who I refer to as BWIs (Black/White individuals)^4^. Using sociolinguistic interview data and self-reported identity markers for participants, as well as a modification of the multiracial identity model proposed by Rockquemore et al. (2008), participants were examined for self-reported identity type as multiracial and/or Black. Participants were recorded in casual peer dyad conversations with friends, and the analysis of their intonational patterns was taken from these recordings. In this study, I found a general pattern such that the participants who identified more as Black, as opposed to multiracial or mixed, were more likely to use a greater quantity of L+H* accents than H* accents. This pattern parallels the findings of McLarty (2018), who found that AAL speakers were more likely to use more L+H*s than MUSE speakers. An example of these accents from this data set, which were also used as stimuli in the current study, can be observed in the Praat (Boersma and Weenink, 2014) spectrograms Figure 1 and Figure 2. In particular, observe the movement of the pitch tracker over the course of the spectrogram.

As we can observe from Figures 1, 2, these intonational contours are differentiated primarily by their shape, with the H* contour simply having a high target, and the L+H* contour having low to high movement all within the prominent syllable.

In addition to the finding that speakers who identified more as Black used a greater quantity of L+H* pitch accent, Author (2016) also found that speakers were more likely to employ L+H* in phrases with narrow, as opposed to broad, focus marking, which is a pattern that would be expected for speakers of MUSE. However, the speakers in Author (2016) who identified most strongly as Black also employed L+H* in broad focus conditions, which is not predicted in MUSE. In English, narrow focus is often thought of as contrastive, and it is characterized by the focused syllable (which must be the stressed syllable in the prominent word) being louder and longer than it would be if the same syllable appeared in broad focus. Though it is difficult to visualize on a spectrogram, focus can be reliably auditorily coded by listeners (Pierrehumbert and Hirschberg 1990). The primary use of narrow focus marking in MUSE appears to be to signal contrast, with speakers having been shown to employ narrow focus more often in situations where they need to indicate contrastive meaning, though this has not been through described or tested in AAL (ibid). Compare, for example, the following phrases, where capital letters are used to indicate narrow focus:

1. Jamal hugged Jim.
2. Jamal hugged LUKE.

Sentence 1 is a common type of phrase with broad focus. However, if we imagine a situation in which a listener hears Sentence 1, knows it to be incorrect, and would like to correct the speaker, the listener may utter Sentence 2, placing narrow focus (realized in part via longer duration and higher amplitude), to provide contrast with the incorrect assertion made in Sentence 1. Though Author (2016) found that L+H* was still likely to occur in such contexts, I also found that the speakers who identified most strongly as Black used L+H* in both broad and narrow focus conditions. While research on MUSE has found L+H* in narrow focus, L+H* in broad focus is not typically predicted, though it is possible that its use specifically in broad focus contexts may be characteristic of AAL.

To date, there is very little research on variation in strategies for focus marking in different varieties of English, though there is some literature indicating that it is theoretically possible since it is a site of variation in other languages. Frota (2002) observes that varieties of European Portuguese (EP) differ from varieties of Brazilian Portuguese in that they employ a specific contour (H*+L) to indicate narrow focus marking. Other languages, including Bengali and Italian, like EP, also use a special pitch accent to cue narrow focus, but languages such as English have not been observed to employ this strategy (ibid, Xu and Xu 2005). Jun and Forman (1996), also posit potential differences between AAL speakers and MUSE speakers with respect to focus marking, though they are not explicit about what these differences may be.

The findings in Author (2016) inspired a number of questions about the perceptual salience of these different pitch contours and focus marking strategies. Using data from a corpus built in that experiment, the current methodology is designed to test the hypothesis that listeners are more likely to rate tokens with an L+H* contour and/or narrow focus marking as having been uttered by a Black speaker. Understanding how these pitch accents and focus marking strategies are produced by speakers and perceived by listeners will help us arrive at a better understanding of the intonational phenomena that may trigger certain types of ethnicity judgments, as well as how these phenomena may be programable to assist computational systems in categorizing user data.

The current study is also unique because of its use of speakers with one Black parent and one White parent (BWIs) as opposed to White speakers and Black speakers. Previous studies on ethnic identification have focused on accuracy as a metric to identify which intonational factors may be salient to listeners, and these methods have generally ignored the rich variation that exists between Black speakers. This is especially limiting, due to the findings of Spears (1988) and Rahman, (2008) indicating that intonational factors may be the most important sites of variation for Black speakers who do not employ stereotyped features of AAL. In a way, this study follows in the path of Lambert et al. (1960) and others since who have employed a matched guise technique, with the primary difference being that the intonational phenomena are what distinguish the guises from one another. Everything else about the speakers’ identities and voices is held constant, so in this way, we may arrive at a more precise understanding of the role of the intonational phenomena itself. This study pushes the field of ethnic identification forward both by using a previously unstudied speaker population, but also by pairing the stimuli that listeners hear specifically by intonational factors.

Following Bryden (1968), Koutstaal and Jackson (1971), Lass et al. (1980), and Thomas and Reaser (2004), this study also addresses the question of how listeners are affected by stimuli that have been altered using a specific type of low pass filter. These studies generally found that listeners were somewhat less accurate at identifying filtered stimuli than unfiltered stimuli, and the current study aims to test this with a new speaker population and as applied to clips that display specific intonational characteristics. As Thomas and Reaser (2004) note, the earlier studies, including their own, have the limitation of not necessarily corresponding to listening conditions in which real people make ethnicity judgments on an everyday basis. Though a lowpass filter may not replicate everyday listening conditions, examining differences between unmodified and filtered results may provide a more comprehensive understanding of how listening conditions affect judgments. The current study, alongside these earlier works, provides further motivation for the careful consideration of listening environment and noise when making claims about how listeners may evaluate speech across a variety of environments that may not resemble the listening conditions of lab speech. Furthermore, understanding both how speakers use these prosodic differences as well as how they are perceived by listeners will inform future research on naturalistic speech recognition and generation that functions more effectively for a wider variety of speakers and users.

## Methodology

### Stimuli

As a result of the fact that intonational variation between AAL and MUSE is still not well documented in the literature, and especially not using modern frameworks for understanding intonational variation, this experiment will act as a first pass at narrowing down the effects of both focus and pitch accent type on filtered and unfiltered speech types, as well as with different groups of listeners organized by race and gender. These results will contribute to our understanding of how certain aspects of ethnolinguistic variation are differentially perceived, and will assist in improving computational systems that necessarily must deal with variable production.

This study uses as stimuli six speakers from Author (2016) and asks listeners to rate the voices under different conditions. The corpus constructed in Author (2016) consists of recordings of young men with one Black parent and one White parent (BWIs), aged 18–32, who were recorded in Washington, D.C. or Eastern Virginia. All of the speakers self-reported that they are native speakers of both MUSE and AAL, though they were never explicitly instructed to speak in one variety or the other, as the original study was designed to explore the speakers” naturalistic range of intonational variation. In these recordings, the speakers are engaged in an “icebreaker”-style game with two different male-identified individuals (one White, one Black) that they identified as close friends. In this game, speakers were instructed to take turns asking each other questions (such as “What’s the worst haircut you’ve ever had?” or “Describe your perfect afternoon”) on cards for 20 min, though only the last 15 min of the recording were analyzed. Author (2016) did not find significant differences in patterns of intonation for these speakers by interlocutor, though I did observe significant differences conditioned by the speakers’ attitudes about race and their own racial identities. The six speakers selected for the current study were the individuals who employed both the L+H* and H* tokens in large enough quantities to create the stimuli needed for the present experimental conditions.

Further information about the speakers' backgrounds, attitudes towards race, and more is available in Author (2016). A more thorough discussion of the speakers’ characteristics is beyond the focus of the current study, though care was taken to select speakers who employed similar patterns of intonational variation to each other in the earlier study, as well as to control for potential speaker effects in the models of analysis. Though it may have been ideal to more tightly control for region, age, or interlocutor, given the limitations of intonational data and the fact that the target pitch accents do not necessarily appear with a high level of frequency for each speaker in each interlocutor condition, it was impossible to do so in the current study. Given the fact that Author (2016) did not find regional, age, or interlocutor differences with respect to the intonational variables of interest in this corpus, it is unclear whether further controlling for these variables in the creation of the stimuli for the current study would yield stimuli significantly different than those employed, since the study is designed primarily to test the perception of different intonational variants. However, replication experiments that employ stimuli from speakers that are more tightly controlled along these dimensions may be a useful avenue for future work.

Intonational phrases from the corpus created by Author (2016) were annotated using the aforementioned ToBI conventions for Mainstream English, in order to obtain phrases with the pitch accents types of interest, H* and L+H* (Beckman et al., 2005). From this corpus, the six speakers who all had phrases of the type of interest were selected. Listeners heard eight intonational phrase-long clips from each speaker in two experimental conditions (one low passed filtered and one unmodified), and the phrases were presented in a randomized order which was different for each listener.

For purposes of this experiment, an intonational phrase was classified as any phrase containing at least one pitch accent and a boundary tone, following the ToBI conventions of Beckman and Ayers (1997). Phrases were selected to be of comparable length (mean syllable length = 6.54), and each phrase contained 2 pitch accents of the same type (either two H*s or two L+H*s), and all ended in the same boundary tone (L-L%). The selection of phrases with two similar pitch accents and an L-L% was made due to the fact that it the stimuli came from naturalistic speech, in which it is difficult to control a priori for the number of pitch accents a speaker may use. Indeed, the combination of two of the same pitch accents and an L-L% boundary tone was the only possible one that was testable given the parameters of the original corpus. As the tokens were extracted from casual speech recordings, it was not possible to control entirely for the semantic content of the phrases, and each phrase was uttered spontaneously in the conversational task context. However, care was taken to avoid tokens that contained explicit discussions of race as well as lexical items that might be associated with AAL (phrases with words such as “dope” and “homie”, which appeared in the corpus, were not included in the stimuli, for example). Additionally, Author (2019) explored the use of 40 segmental phonological and morphosyntactic features of AAL in the same corpus, and found that they occurred extremely infrequently (mean N = <8 occurrences/20 min task), thus somewhat mitigating possible effects of these other types of features. Of course, there are a number of additional phonetic features that may correlate with AAE which could influence such judgments, though they were beyond the scope of the current analysis. Though the use of casual speech creates some unique limitations, it has the advantage of being naturalistic and therefore may be more appropriate for testing how listeners may make ethnicity judgments in realistic situations.

With respect to focus, Author (2016) labelled the phrases in the corpus as having broad or narrow focus, based on syntactic, semantic, and phonetic criteria. The phrases tested in this experiment are taken directly from that data set, with their corresponding focus labels. The combinations of pitch accents and focus marking in the stimuli, along with example phrases, appear in the Table 1 below. This table contains all of the combinations of variables of interest that the listeners heard from one speaker in order to provide the reader with greater clarity about the experimental design.

**TABLE 1:** Stimuli set for one speaker with 8 clips under the 4 different possible intonational conditions. In narrow focus conditions, the word where the narrow focus appears is indicated in bold. Example phrases from one participant with narrow focus lexical items in bold.

Clip	PA Type	Focus Type
of thirty-one years	H*	B
livin’ in that house	H*	B
if she went to school or not	H*	N
then I think I would	H*	N
and even before that	L+H*	B
four days off	L+H*	B
and it was a union job	L+H*	N
thing that I can imagine	L+H*	N

The experiment was designed in this fashion in order to allow of number of different direct comparisons during the analysis phase. These comparisons were as follows:

1. The effect of H* vs. L+H* regardless of focus.
2. The effect of broad vs. narrow focus, regardless of pitch accent type.
3. The effect of low-pass filtering vs. original clips on ratings, independent of intonational contours and focus.
4. The effect of low-pass filtering vs. original clips on ratings as a result of broad vs. narrow focus and/or H* vs. L+H* pitch accent types.

#### a. The Experiment

45 listeners were recruited via a university participant pool and well as through friend-of-a-friend methods. The listeners all identified as Black or White and as male or female, and the sample was balanced to obtain comparable numbers of listeners from each gender/race pairing^5^. Listeners were primarily undergraduates at a large, private university and the experiment was conducted in a quiet room in a university’s phonetics laboratory. Upon arrival, listeners were instructed that the experiment would proceed in two parts. Listeners were outfitted with a pair of Bose headphones, and then followed the experiment in an online survey hosted by Qualtrics. They read and agreed to a consent form and then heard a sample sentence for which they were asked to decide whether the speaker was Black or White. After that, they began experiment Task 1. In Task 1, listeners heard 48 clips in a randomized order (8 from each speaker, and counterbalanced for focus and pitch accent type variables and following each clip), and were asked to respond to the binary choice question “What is the ethnicity of the speaker” as quickly as possible. In Task 1, the clips were low pass filtered at 400 Hz, following Knoll et al., 2009, in order to obscure most segmental information but retain F0 information (Thomas and Reaser 2004). Listeners were instructed to open the door and alert the researcher when they completed Task 1. The researcher then entered the room and confirmed that the participant had reached the end of Task 1.

In Task 2, the listeners repeated the same task, but this time they heard the original unmodified versions of the same phrases. Following Tasks 1 and 2, the listeners were asked the following series of questions about the experiment:

1. What did you think of the tasks?
2. How easy or difficult or easy was the task?
3. How many different voices do you think you heard in each part?
4. Do you have any other comments about the experiment?

Following these questions, the participants were also asked a series of open-ended demographic questions including their gender, age, level of education, race, places of residence during their lifetime, and linguistic ability in languages other than English. This data was examined qualitatively to check for broad patterns related to listener experience. In the end, participants were age 20–30 and either current university students or recent graduates, so age and education were not variable enough to test for listener differences. From the qualitative analysis, which was necessary due to the small data set, there were also no clear patterns with respect to region or L1 experience. The regression models do include gender and race as factors however, since these were the only factors that could be included in the model and still yield functional results.

## Analysis

The analytical methods employed were designed to examine whether pitch accent type, focus type, or their interaction affected listener judgments of ethnicity by comparing clips in which these factors varied reliably. They were also designed to control for aspects of listener demographics, such as gender and race and the interaction thereof. Multiple logistic regression models were conducted in R using the lme4 (Bates et al., 2015) package and plotted using ggplot2 (Wickham 2009), and the results of the regression models are presented in the tables in Appendix A (Task 1) and Appendix B (Task 2). Both models presented here controlled for the effects of speaker and subject as random effects in order to mitigate the effects of individual variation, and since each speaker uttered different tokens, the effects of utterance are also partially controlled, though it was impossible to include token as a random effect due to the relatively small size of the stimuli set. The results for the clips in Task 1 and Task 2 appear to differ substantially, as well as indicate that speakers may be using a different decision-making process for these clips. As a result, the analysis for these two tasks will be presented independently, with discussion and comparison of the two tasks to follow.

### Task 1: Filtered Clips

In Task 1, listeners were presented with a Qualtrics survey that contained 48 clips filtered at 400 Hz, building on the methods discussed in Thomas and Reaser (2004) and Knoll et al. (2009). A filtering condition of 400 Hz was chosen to maintain features of F0 while obscuring the majority of formant and segmental information. These clips were counterbalanced for the variables of pitch accent type, focus type, and interaction, as presented in Table 1 above. Listeners were presented with each clip and then instructed to respond to the forced choice question “What is the ethnicity of this speaker?” and given the options of “Black” or “White”. They were instructed to respond to this question as quickly as possible.

**FIGURE 3:**
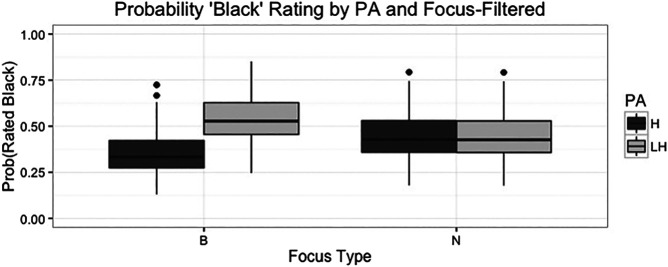
Boxplot showing likelihood of stimuli being rated ‘Black’ based on pitch accent ratings, focus type, and the interaction for the filtered listening condition. The bars on the left show that the combination of L+H* and broad focus is more likely to be rated as “Black” than the combination of H* and broad focus. The bars on the right show no difference between ratings of the two different pitch accent types in the narrow focus conditions.

The logistic regression model fitted for this task was (Response∼SubjectGender*SubjectRace+PA*Focus+(1|Speaker)+(1|Subject), family=binomial). Results of this model examining listener responses with pitch accent type, focus type, listener gender, and listener race as fixed effects, and speaker and listener as random effects reveal a significant main effect for pitch accent type, indicating that the stimuli with the L+H* pitch accents were significantly more likely to be labeled as having been uttered by a Black speaker (*p* < 0.001), as can be observed in Figure 3. In contrast, the results reveal no significant main effect of focus on likelihood of being rated as having been uttered by a Black speaker.

However, there is also an interaction between focus and pitch accent type, such that clips with the combination of broad focus and L+H* contours are more likely to be rated as Black (*p* < 0.001) though there is no significant difference between pitch accent types in the narrow focus condition. Figure 3 shows these results for the main effects as well as this interaction.

As we can observe from Figure 3 above, in the narrow focus condition on the right, pitch accent does not appear to affect the probability of a clip being rated “Black”. However, in the broad focus condition on the left, clips with the L+H* pitch accent are significantly more likely to be rated as having been uttered by a Black speaker.

The regression model controlled for speaker and subject as random effects, in order to ensure that these observed differences were not primarily driven by the ratings of a particular speaker or listener subject. Finally, perhaps surprisingly, no significant results were obtained in the analysis of listener race/gender or the interaction of these variables, indicating that listener judgments in this task do not appear to be subject to variation based on those aspects of listener identity, in contrast with the results obtained in earlier studies by Foreman (2000) and Thomas and Reaser (2004).

### Task 2: Unmodified Clips

In Task 2, which immediately followed Task 1, listeners were presented with another Qualtrics survey that contained 48 randomized clips, but in this condition, the clips were in their original, unmodified versions. These clips were identical to the clips in Task 1 except that they were unfiltered, and so were also counterbalanced for the variables of pitch accent type, focus type, and interaction, as discussed above. Listeners were again presented with each clip and then instructed to respond to the forced choice question “What is the ethnicity of this speaker?” and given the options of “Black” or “White”. They were instructed to respond to this question as quickly as possible.

**FIGURE 4:**
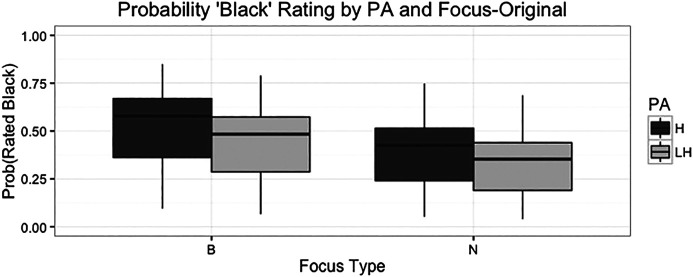
Boxplot with error bars showing pitch accent ratings by focus type for the original stimuli listening condition. The bars on the left show that the H* accent is more likely to be rated as “Black” in the broad focus condition. The bars on the right show the same pattern for the narrow focus type condition, indicating no interaction of pitch accent and focus in this condition.

The logistic regression model fitted for this task was identical to the one fitted for task 1, except for now it was run on responses unfiltered stimuli. The formula was Response∼SubjectGender*SubjectRace+PA*Focus+(1|Speaker)+(1|Subject), family=binomial. Results of this logistic regression model examining listener responses with focus type, pitch accent type, subject gender, and subject race as fixed effects, and speaker and listener as random effects reveal that pitch accent type is a significant factor such that clips with the L+H* are less likely to be labeled as having been uttered by a Black speaker (*p* < 0.001). With respect to the question of focus, we also obtain a significant effect such that stimuli with the narrow focus were also less likely to be labeled as Black (*p* = 0.00273). Figure 4 shows these results.

These results represent somewhat of a reversal of the trends observed for the responses in Task 1, though unlike in Task 1, the interaction of the two variables was not significant. Overall, broad focus tokens were less likely to be rated as “Black”, as were those with the L+H* pitch accent. The regression model again controlled for the effects of speaker and subject, in order to ensure that these observed differences were not primarily driven by the ratings of a particular speaker or listener subject. Again, no significant differences were observed between groups of listeners organized by race, gender, or the interaction.

### Comparing Tasks

In general, for the main effects, we can observe contrasting patterns for listener ratings between the filtered and original listening conditions, which is a result not previously documented in studies of ethnic identification. Additionally, we observe some interactions between pitch accent type and focus type with respect to the ratings. Table 2A below synthesizes the main effect results obtained in the previous two sections.

**TABLE 2A:** Results for main effects of pitch accent type as well as focus type in each listening condition.

Variable	Filtered Condition (judged as)	Original Condition (judged as)
H*	White	Black
L+H*	Black	White
Broad Focus	None	Black
Narrow Focus	None	White

As we can observe from this table, the ratings (probability of rated “Black”) pattern in opposite directions for the filtered and original listening conditions for the two different types of pitch accents. While focus does not appear to act as a main effect influencing ratings in the filtered condition, it is significant in the original condition. The table below synthesizes the interaction results obtained in the previous two sections.

As is evident from the results the Table 2B above, the effects pattern in opposite directions for the filtered vs. the original clips with respect to the interaction of pitch accent and focus. This result stands in contrast to the results of other studies which have found that low-pass filtering causes listeners to be less accurate in their judgments or to simply behave at chance for filtered segments (Bryden 1968; Lass et al., 1980; Thomas and Reaser 2004).

**TABLE 2B:** Results for interaction effects of pitch accent type as well as focus type in each listening condition.

Interaction Variables	Filtered Condition (judged as)	Original Condition (judged as)
L+H*+Narrow	None	White
H*+Narrow	None	Black
L+H*+Broad	Black	White
H*+Broad	White	Black

Finally, with respect to potential task effects, it does not appear that listeners were subject to training effects of the tasks, though it is important to note that all participants heard the filter clips before the unmodified clips. This design was intended to prevent training effects, given that clips with more segmental information may be more easily recognizable that clips with less such information, though it does present a limitation of the current study since results can only be interpreted given this testing order. During the experiment debrief, listeners were asked to report the number of voices that they thought they heard across the two tasks. If listeners were indeed responding to a training effect, we may expect that they would report hearing a lower number of voices than what they actually heard. The mean number of voices reported for the listeners who was 8.31, though 7 out of the 43 listeners responded, “I don’t know”, indicating that over 16% of the sample was uncomfortable guessing how many voices were in the stimuli. This provides some evidence against a noticeable training effect, as does the fact that stimuli were randomized for each speaker in each task. Future studies, however, should consider additional methods for randomizing stimuli presentation in order to further test for such effects.

## Discussion

The results of this study which tested listeners’ ratings of clips as “Black” or “White” under two listening conditions, original, and low pass filtered, while controlling for specific intonational phenomena of pitch accent type (L+H* vs. H*) and utilizing clips that had broad vs. narrow focus, yielded results that show that listeners appear to interpret these intonational phenomena in different, sometimes opposite, ways in filtered vs. original listening conditions. Previous studies on ethnic identification have generally found that listeners were less accurate under filtered conditions, but to date, this is the first study that has found that listeners may actually judge filtered and unfiltered clips in significantly different ways. By controlling for intonational contours as well as focus type, we have observed that listeners may differentially interpret the effects of intonational contours based on listening condition when attempting ethnic identification. This finding is particularly important for computational applications, especially ASR and speech synthesis systems, because if meaning of a particular contour is context-dependent for listeners, accurate systems must also take this into account. When attempting to accurately synthesize the speech of a Black speaker such that a listener would receive accurate sociolinguistic information, systems would necessarily have to account for how listeners make different judgments depending on acoustic quality. This is of particular importance since Black speakers have been historically underserved by computational systems; as Blodgett et al. (2020) note: “in the technology industry, speakers of AAE are often not considered consumers who matter” (9). This study thus provides important sociolinguistic context for computational researchers who aim to address this inequality.

This study also differs from previous studies in that its aim was not to test accuracy, but rather specifically examine differences between the effects of pitch accents, focus type, and filtering on listener judgments thus providing greater utility for computational applications. Given the results of McLarty (2018) and Author (2016) which have shown that AAL speakers may be more likely to employ the L+H* pitch accent than MUSE speakers, especially in broad focus contexts, one might expect that listeners would be consistently more likely to rate the L+H* pitch accent as having been uttered by a Black speaker. Additionally, (Author 2016), found that BWI speakers showed a pattern such that those who identified as more Black were more likely to use L+H*, and that this was especially the case in broad focus conditions. However, a consistent relationship between these patterns of production and perception was not obtained for the clips in the original stimuli in this study. Listeners appear more likely to judge that contour as “Black” only in low-pass filtered condition and not when they hear the original stimuli. These results also indicate that speakers’ interpretation of intonational variables can differ depending primarily upon how much linguistic information they have available to them. That is, when speakers were exposed to filtered speech, hearing the L+H* pitch accent caused them to be more likely to rate the voice as Black. Interestingly, however, the interaction of narrow focus and L+H* gets rated as LESS Black, perhaps due to differences in salience and meaning of that pitch accent between MUSE and AAL (Thomas 2015, Author 2016). In particular, since earlier research has found that MUSE listeners may expect L+H* to signal contrastive meaning, it may be more marked in situations where it does not perform that function, such as in phrases with broad focus (Pierrehumbert and Hirschberg 1990; Watson et al., 2008). Indeed, for both ASR and synthesis, capturing such ethnolinguistic different in prosodic contour meaning will be important for addressing not only user experience, but also bias in systems. Given that ASR is increasingly used for a variety of purposes, understanding whether or not a particular syllable has a pitch accent that signals contrastive focus may be important for the interpretation of the meaning of entire phrases.

Additionally, there is the possibility that the meaning of the L+H* pitch accent may differ between ethnolinguistic varieties, and also therefore potentially influence listener judgements. While the current study did not observe systematic differences with respect to judgments related to listener race, information about how individual listeners interpret the meanings of these intonational contours may further shed light on the mechanisms by which speakers make ethnicity judgments. As we have observed in earlier studies, especially Foreman (2000) and Thomas et al. (2010), Black and White listeners do sometimes pattern differently in ethnic identification tasks. Future studies should specifically address the potential for differential interpretations of the ethnolinguistic meanings of specific intonational contours for groups of listeners with different demographics. If the L+H* pitch accent sounds generally more marked/less standard for many listeners, synthesis systems must learn to employ pitch accents and boundary tones in a naturalistic way for voices that may be designed to represent different ethnolinguistic backgrounds.

Previous studies on ethnic identification have found that listeners may attune to a number of segmental and suprasegmental features in making ethnicity judgments, but that intonational variation does seem to play a significant role (cf Thomas et al., 2010). While the current study controlled for random effects of speaker and subject, it was unable to control for segmental phonological features due to the fact that it employed naturalistic speech. Intonation studies often face a difficult task of balancing the desire for control of segmental and syntactic information with the desire for naturalistic speech, and so it is possible that some features which were not entirely controlled for in the current study may also interact with the results obtained. Future work could compliment the results obtained here by using read speech, though that introduces a complication related to prosodic naturalness. However, comparing the results of studies that examined naturalistic vs. controlled speech might better shed light on these possible effects. The interaction between prosodic and segmental phonological variables will also be important for both ASR and speech synthesis systems, given that they also frequently rely on naturalistic speech. In particular, though systems may be improving in the naturalness of the production of segments, failure to replicate naturalistic prosody, or to combine naturalistic segments and contours will also limit improvements in speech synthesis.

These findings may also have broader consequences for linguistic profiling, which can have negative impacts on speakers’ educational opportunities, economic prospects, as well as other types of interactions with government systems (Baugh 2003, 2015). Though the research has primarily focused on the ways in which stigmatized segmental and grammatical features may influence profiling, the fact that intonation is salient for listeners means that linguists need much more information on how listeners and speakers perceive and employ intonational variation at every level (Thomas 2015). Teaching speakers and listeners as well as communities to recognize the linguistic variables that may affect their perception of certain voices may be an important first step towards mitigating the often unconscious effects of linguistic profiling. With respect to ASR systems, a better understanding of the mechanisms by which this type of linguistic profiling occurs may also prevent future systems from miscategorizing or misevaluating the speech of user who employ non-standard varieties, thus creating a more equitable user experience. For speech synthesis, comprehensive models that rely on replicating naturalistic variation at all levels of linguistic structure will better serve individuals from a variety of background, thus improving their user experience and quality of life.

## Data Availability

The raw data supporting the conclusions of this article will be made available by the authors, without undue reservation.

## Footnotes

1 I used AAL here as a cover term for several varieties of English spoken in Black American communities, following Lanehart (2015).

2 To a modern reader, these may be colorfully dated phrases. “None of that off the wall stuff”, “What’s happ’nin man”, “Man I don’t play the dozens,” “Let’s go grease,” and “She ain’t nothing but a stone fox”.

3 It is unclear whether any of their contours correspond directly to the ToBI H* or L+H* employed in the current study.

4 Black/White Individuals. Participants in this study self-identify with a variety of racial categories, but it is important to note that the speakers in this sample initially only responded affirmatively to the question “do you have one Black parent and one White parent?” in the recruitment phase. For this reason, I have chosen to discuss them only by their response indicating their parentage, which allows us to discuss their external societal classifications and ancestries without ignoring their individual and nuanced self-descriptions as “multiracial”, “biracial”, “Black”, “White”, “other” or any combination thereof.

5 The sample contained 10 White men, 10 White women, 10 Black men, and 15 Black women. Participants from other racial/ethnic groups were excluded due to a lack of clarity in earlier literature about the perceptions of MUSE and AAL among groups of non-Black and non-White individuals.

## Appendix

### A. Full Text of Stimuli Sentences.

**Table:**

Speaker	Clip	PA Type	Focus Type
1	of thirty-one years	H*	B
1	livin’ in that house	H*	B
1	if she went to school or not	H*	N
**1**	then I think I would	H*	N
1	and even before that	L+H*	B
1	four days off	L+H*	B
1	and it was a union job	L+H*	N
1	thing that I can imagine	L+H*	N
2	to do something	H*	B
2	out of my mind	H*	B
2	at the store	H*	N
2	the second thing	H*	N
2	in my headphones	L+H*	B
2	after a while	L+H*	B
2	I was terrified	L+H*	N
2	now this is getting personal	L+H*	N
3	I thought were pretty	H*	B
3	being around	H*	B
3	terrified of heights	H*	N
3	you have to pass	H*	N
3	I would have	L+H*	B
3	really understand	L+H*	B
3	the college mentality	L+H*	N
3	in the long run	L+H*	N
4	close at like eleven	H*	B
4	that movie actually	H*	B
4	that you talk to girls	H*	N
4	that's why I hated going to work	H*	N
4	I don't really	L+H*	B
4	it was really cold	L+H*	B
4	have a job fair	L+H*	N
4	and even more fun	L+H*	N
5	I would say	H*	B
5	but not empowered in	H*	B
5	all these companies that	H*	N
5	being in the park	H*	N
5	and there you go	L+H*	B
5	come to africa	L+H*	B
5	in the bank	L+H*	N
5	after the show	L+H*	N
6	because of just	H*	B
6	I had a great time	H*	B
6	I did nothing	H*	N
6	getting attacked	H*	N
6	this woman got	L+H*	B
6	I don't know	L+H*	B
6	that's a good one	L+H*	N
6	three in the morning	L+H*	N

### B. Regression Table for Task 1 (Filtered Clips) With Black/White as Response Variable, plotted using stargazer package (Hlavac 2015)

**Table:**

	*Dependent variable:*
	Response
SubjectGenderM	−0.216
	(0.191)
SubjectRaceW	0.091
	(0.192)
PALH	−0.779^***^
	(0.126)
FocusN	−0.379^***^
	(0.126)
SubjectGenderM:SubjectRaceW	−0.410
	(0.285)
PALH:FocusN	0.787^***^
	(0.178)
Constant	0.749^***^
	(0.216)
Observations	2,254
Log Likelihood	−1,474.723
Akaike Inf. Crit.	2,967.446
Bayesian Inf. Crit.	3,018.930

*Note*: **p* < 0.1;***p* < 0.05; ****p* < 0.01.

### C. Regression Table for Task 1 (Unmodified Clips) With Black/White as Response Variable, plotted using stargazer package (Hlavac 2015)

**Table:**

	*Dependent variable*
	Response
SubjectGenderM	−0.267
	(0.227)
SubjectRaceW	0.042
	(0.228)
PALH	0.405^***^
	(0.135)
FocusN	0.642^***^
	(0.136)
SubjectGenderM:SubjectRaceW	−0.212
	(0.339)
PALH:FocusN	−0.103
	(0.191)
Constant	0.085
	(0.359)
Observations	2,162
Log Likelihood	−1,311.175
Akaike Inf. Crit.	2,640.350
Bayesian Inf. Crit.	2,691.459

*Note:* **p* < 0.1; ***p* < 0.05; ****p* < 0.01.
